# An Investigation into the Poor Survival of an Endangered Coho Salmon Population

**DOI:** 10.1371/journal.pone.0010869

**Published:** 2010-05-28

**Authors:** Cedar M. Chittenden, Michael C. Melnychuk, David W. Welch, R. Scott McKinley

**Affiliations:** 1 Department of Arctic and Marine Biology, University of Tromsø, Tromsø, Norway; 2 The University of British Columbia Centre for Aquaculture and Environmental Research, West Vancouver, British Columbia, Canada; 3 Fisheries Centre and Department of Zoology, The University of British Columbia, Vancouver, British Columbia, Canada; 4 Kintama Research Corporation, Nanaimo, British Columbia, Canada; Freie Universitaet Berlin, Germany

## Abstract

To investigate reasons for the decline of an endangered population of coho salmon (*O. kisutch*), 190 smolts were acoustically tagged during three consecutive years and their movements and survival were estimated using the Pacific Ocean Shelf Tracking project (POST) array. Median travel times of the Thompson River coho salmon smolts to the lower Fraser River sub-array were 16, 12 and 10 days during 2004, 2005 and 2006, respectively. Few smolts were recorded on marine arrays. Freshwater survival rates of the tagged smolts during their downstream migration were 0.0–5.6% (0.0–9.0% s.e.) in 2004, 7.0% (6.2% s.e.) in 2005, and 50.9% (18.6% s.e.) in 2006. Overall smolt-to-adult return rates exhibited a similar pattern, which suggests that low freshwater survival rates of out-migrating smolts may be a primary reason for the poor conservation status of this endangered coho salmon population.

## Introduction

**Figure 1:**
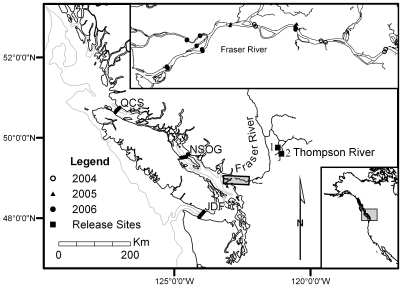
Geographic location of part of the POST acoustic array and smolt release sites. The edge of the continental shelf (200 m depth contour) is shown, as well as acoustic listening lines located in the Fraser River, Strait of Juan de Fuca (JDF), the northern Strait of Georgia (NSOG), and the Queen Charlotte Strait (QCS). Thompson River coho salmon smolts were released at 1) Spius Creek and 2) the Coldwater River.

Many salmon populations are in decline worldwide [1]–[3], with low marine survival identified as the primary cause [4], [5]. In this study we report specific results that suggest a freshwater problem during the coho smolt migration is also a contributing factor in the Thompson River system of British Columbia (BC; Fig. 1).

Genetically distinct from coho salmon (*Oncorynchus kisutch*) populations in the lower Fraser River and the rest of BC, Thompson River coho salmon (Thompson coho) are closely related to the extinct upper Columbia River coho populations [6]–[8]. Returns of wild Thompson coho were relatively stable during the 1970s, and increased during the 1980s [3]. However, between 1988 and 2000, the Thompson coho population declined by 90%, making it one of Canada's most endangered salmon populations [3], [9]. An unprecedented moratorium on the west coast salmon fishery was implemented in 1998, but the Thompson coho population did not recover [10]. Thus over-fishing alone did not prevent the recovery of the population. An urgent request for investigations into the cause of the Thompson coho's demise was made shortly thereafter by the government of Canada [11].

The overall marine survival of coho salmon in southern BC has declined during the past three decades [10], [12]–[15]. When a regime shift occurred in 1989–1990, ocean productivity decreased in southern BC and smolt-to-adult marine survival of both coho [16], [17] and steelhead trout (*O. mykiss*) plummeted [1], [2]. Four Canadian salmon populations became endangered after 1989, including the Thompson coho [18]. Coho salmon production off the west coast of North America has varied with latitude, however. While the southern populations dwindled, northern populations of coho salmon and steelhead trout had record-high abundances [2], [19], [20].

In addition to altering salmonid marine survival rates, ocean conditions have been correlated to changes in the migratory pattern of Strait of Georgia coho populations [4], [16] (which the Thompson coho migrate into after exiting the Fraser River) and other salmon species [21]–[24]. The Strait of Georgia once supported large commercial and recreational coho fisheries (valued at CDN $218.5 million [25]). During the mid-1990s the Strait of Georgia coho fishery collapsed, likely due to a decline in marine survival and a complete shift in migratory behaviour [16]. The Thompson coho, one of the primary Strait of Georgia coho populations, historically spent the marine phase of their life cycle almost entirely within the Strait of Georgia [26]. Since 1995, however, nearly all of the coho left the Strait by the February of their first ocean winter [27]. The cause of this behavioural change is not known, but as with most recent changes in salmon survival and behaviour, the leading hypothesis attributes it to shifting ocean productivity [4]. The Strait of Georgia has had an increasing average sea surface temperature as well as other oceanographic trends attributed to the changing climate [4].

In addition to the trends observed in the marine environment, freshwater productivity and habitat quality are also affected by changes in climate [28]. However, there is a lack of quantitative evidence concerning the effects of climate change on the freshwater production of coho salmon smolts [29]. Recent climate trends in the North Pacific have been correlated to higher temperatures and earlier spring flows in the Fraser River system [30], which seems to be advancing the out-migration timing of wild coho smolts [27]. In the Thompson River system, an increasing average April flow rate [31]—indicating an earlier spring freshet—may be affecting the early freshwater survival and behaviour of wild and hatchery Thompson coho smolts. Decreased water quality during high flow periods [31] is also a major concern for coho salmon in the Thompson watershed. Rood and Hamilton [32] found that during the summer months in this semi-arid valley, large amounts of water are being withdrawn from the Thompson River for irrigation purposes, lowering flows and increasing mean summer temperatures. As coho spend their first year in the river environment, they are especially sensitive to flow levels, temperature extremes, siltation, predation, and disease [33]. Optimal survival conditions for coho smolts were found to be in rivers with relatively cooler temperatures, deep pools, structurally complex habitats, intermediate second winter flows, and high second spring flows [34]. Fluctuations in the abundance of Thompson coho were weakly correlated to agricultural land use, road density and stream habitat quality [10]. Concerns have been expressed repeatedly about the freshwater habitat quality in the Thompson River watershed, as well as the need for coho riverine-survival data that could provide evidence linking weak populations to freshwater habitat concerns [9], [11].

Over-fishing, changes in ocean climate and a basin-wide deterioration of freshwater habitat are believed to be the primary causes of the Thompson coho decline [10]. To investigate smolt survival rates as well as the migratory behaviour of Thompson coho smolts, hatchery-reared fish were tagged during three consecutive years (2004–2006) and monitored by the Pacific Ocean Shelf Tracking project (POST) array [35]. The objective of this study was to identify areas of high mortality for this population during their early migration phase, with the goal of providing a focus for conservation efforts. A two-year tag effects study was carried out concurrently to evaluate the post-surgical growth, survival, tag retention, health and swimming ability of acoustically tagged smolts [36].

## Methods

### Ethics Statement

All work involving live fish reported in this paper was annually reviewed and pre-approved as meeting or exceeding the standards laid out by the Canadian Council on Animal Care. In 2004 and 2005 the project guidelines were approved by the Department of Fisheries and Oceans Canada Pacific Region Animal Care Committee. In 2006 review and approvals were made by the Animal Care Committee of Malaspina University-College (now Vancouver Island University), c/o Patricia Stuart, Vancouver Island University, Nanaimo, BC, Canada.

### Study Area

Near the end of the last ice age approximately 15,000 years ago, the Fraser River canyon was blocked by ice, forcing the upper Fraser and Thompson Rivers to drain southward into the Columbia River. This provided the opportunity for many species, including coho salmon, to colonize the Thompson River from the Columbia River refugium [37]. The Fraser canyon continues to act as a velocity barrier to many fish species and populations, dividing the Upper and Lower Fraser River into distinct habitat zones and genetically distinct coho populations. Genetic data indicate that there has been almost no interchange between upper and lower Fraser River coho populations during the past 10,000 years [38]. Thompson coho populations can be divided into three sub-regions: the North Thompson, the South Thompson and the Lower Thompson/Nicola. The populations studied here were from the Lower Thompson/Nicola group.

The 1,370 km long Fraser River (Fig. 1) is the largest river in BC, with a watershed of 233,100 km^2^, and an average yearly flow rate of 3,540 m^3^·s^−1^. The Thompson River, at 489 km long, is the largest tributary of the Fraser River, with a watershed of 55,400 km^2^. Water quality and flow rate monitoring of the Thompson River began in 1911 at the Spences Bridge station, 40 km upstream from where the Thompson River enters the Fraser River. Average annual flow rates during 2004, 2005, and 2006 were 672, 784 and 656 m^3^·s^−1^ respectively, which fall near the 1912–2006 annual average of 765 m^3^·s^−1^ [31]. Peak flow rates were 1,820 m^3^·s^−1^ on 4 June 2004, 2,230 m^3^·s^−1^ on 19 May 2005, and 2,630 m^3^·s^−1^ on 27 May, 2006. Low flow rates were 171 m^3^·s^−1^ on 4 March 2004, 250 m^3^·s^−1^ on 17 June 2005, and 193 m^3^·s^−1^ on 28 October 2006 [31]. The timing of the spring freshet in the Fraser River has been advancing during the past century [16], and a similar trend has been observed in the Thompson River [31].

The Thompson River water quality is well-buffered and soft, with pH and oxygen levels “within normal ranges for aquatic life” [39]. However, levels of non-filterable residue, turbidity, total aluminum, iron and phosphorus often exceed recommended levels for fish during the spring freshset [31]. There was an increasing trend from 1973–1997 in dissolved chloride and copper [39]. The minimum detectable limit for copper, however, was higher than the limit for aquatic life (0.002 mg·L^−1^) [31]. Non-filterable residue and turbidity level increases have been attributed to increased agriculture, forestry and residential development [39]. The temperature of the South Thompson River during the spring freshet is generally not a problem for salmon [31]. However, the mean summer temperatures of the entire Fraser River system have been increasing over the last century; during the next 100 years, the potential for salmon to be exposed to temperatures higher than 20°C is predicted to increase by a factor of ten [40].

Most Thompson coho salmon spend their first year-and-a-half in freshwater and the following two years in the ocean, before returning to their natal streams to spawn [10]. Hatchery production was initiated in the Thompson watershed in the early 1980s to test enhancement strategies for coho [41], [42]. Smolt production began at the Spius Creek Hatchery in 1984 to rebuild depressed populations. Assessments of returns for both wild and hatchery populations were carried out regularly thereafter. The hatchery coho broodstock is collected from the wild population each year.

### Surgical Protocols and Smolt Releases

Coho smolts from two different tributaries—the Coldwater River (2004, 2006) and Spius Creek (2005)—were tagged at the Spius Creek Hatchery using previously established protocols [43], [44]. During 2004, 40 Coldwater River coho smolts from the 2002 brood were implanted with V7-2L acoustic transmitters (12 fish; 7×18.5 mm, mass in air 1.4 g, mass in water 0.7 g, frequency 69 kHz, 30–90 s random delay, VEMCO Ltd, Halifax, Nova Scotia Canada), and V9-6L acoustic transmitters (28 fish; 9×20 mm, mass in air 3.3 g, mass in water 2.0 g, frequency 69 kHz, 30–90 s, VEMCO Ltd, Halifax, Nova Scotia Canada). The surgeries were carried out 30 May 2004. The average fork length (±SD) of the fish tagged with 7 mm tags was 128±1 mm (range 127–129 mm), and for those with 9 mm tags, 132±2 mm (130–141 mm). The river temperature was 11.5±0.6°C during the surgeries. The release dates of 88,300 hatchery coho smolts were from 19–22 May 2004; the tagged fish were released 31 May 2004 at 1400 PDT in the Coldwater River (Fig. 1). For a comparison of hatchery and wild smolt-to-adult survival rates of Pacific salmon, see Walters and Ward [45].

During 2005, 50 Spius Creek coho smolts from the 2003 brood were implanted with V7-2L tags. The river temperature averaged 9.0±0.7°C on the day of the surgeries, 17 May 2005. The smolts had an average fork length of 128±4 mm (125–139 mm). They were released on 19 May 2005 at 1115 PDT in Spius Creek to coincide with the hatchery release of 58,450 coho smolts.

The 2004 brood of the Coldwater River population was tagged in 2006 from 25–26 May with V7-2L transmitters. One hundred smolts were implanted with V7-2L transmitters and released 29 May 2006, at 1300 PDT. The average fork length of the tagged smolts was 130±3 mm (125–141 mm). The river temperature during the surgeries was 8.2±0.9°C. The other hatchery releases were as follows: 43,000 smolts were released 9 May 2006, 20,000 smolts were released 26 May 2006, and 6,460 smolts were released 29 May 2006 in the Coldwater River (Fig. 1) at the time of the release of the tagged smolts.

Tag effect studies on 500 Coldwater River coho smolts during 2005 and 2006 demonstrated that the implantation of V7-2L tags did not impact fish survival or swimming ability at the body sizes used in the field study when compared with control groups [36]. Physiological assessments, swimming performance and growth were all similar to control values [36]. Smolts with an initial fork length ≥125 mm (the minimum size used in this study) tagged with V7-2L transmitters had 100% tag retention and survival over a 300 day period [36]. The larger V9-6L tags implanted in coho smolts of the size range tagged in the field study (130–141 mm) had 92–100% survival and tag retention three weeks after tagging [36]. Monitoring of the achieved life-span of V9-6L and V7-2L tags deliberately held back and monitored in the laboratory indicates essentially 100% operation to 90 days post-activation (Welch, unpublished data), which is far longer than the migration times measured in this study (ca. two weeks).

### Acoustic receiver array

Acoustic receivers (models VR2 and VR3, VEMCO Ltd, Halifax, Nova Scotia Canada) were located both in the Fraser River and in the ocean (forming the POST array; Fig. 1) to track the smolts' downstream migration. A summary of the Fraser River sub-line locations is available in Welch et al. [46]. Briefly, the total distance from the release site to the mouth of the Fraser River is approximately 385 km from the 2005 release site and 420 km from the 2004 and 2006 release sites. Distances from release sites to the last detection station in the Fraser River were 381 km, 353 km and 410 km during 2004, 2005 and 2006, respectively. During 2004, there were six receivers in the Fraser River arranged in three lines of paired units from April to August, then five receivers from August to November. Four receivers (two lines of paired units) were deployed in the Fraser River in April of 2005 and recovered in December. Eighteen receivers were deployed in the Fraser River from April to December of 2006. These were arranged in three main lines, where the lower two lines contained two or three sub-lines each to cover multiple channels of the braided lower river (Fig. 1).

In ocean waters, acoustic receivers were positioned to form sub-lines extending across the northern Strait of Georgia, Queen Charlotte Strait, Juan de Fuca Strait, and Howe Sound (Fig. 1). Also, in 2006, receivers were moored in Burrard Inlet and the southern Strait of Georgia. During 2005 and 2006, three receivers operated by the Vancouver Aquarium were located at Point Atkinson (Burrard Inlet and Point Atkinson are located in the ocean near the Fraser River mouth; for exact locations, see www.postcoml.org).

### Data Analysis

We compiled a database of detections from acoustic receivers consisting of the time and location where an individual tag was detected. First, we identified a list of suspect detections likely to be false positives due to tag code collisions (when the signals from two or more tags interfere to cancel each other out or create false tag codes) or other noise sources. Detections of fish were excluded as false if they were detected only once on a line within 60 minutes, had one or more tags heard on the same receiver around the time of the suspect detection, and did not have supporting detections from other time periods or lines. Supporting detections are defined as a temporal sequence of detections from the release date along the migration path. After eliminating the suspect detections, we used these filtered data to estimate survival and detection probabilities as well as the travel times of tagged smolts during the downstream migration. Travel times in each segment were measured as the difference between successive lines in the cumulative travel times from release until the first detection of a tag on a line. Median travel times were calculated as the linearly interpolated time at which 50% of the survivors reached a detection point.

### Survival probability estimation

We used variations of the fully time-varying Cormack-Jolly-Seber (CJS) mark-recapture model for live recaptures to estimate survival probabilities (φ) in each segment of the downstream migration [47]–[49]. This model simultaneously estimates detection probabilities (*p*) at each line of receivers in the Fraser River and adjusts survival estimates accordingly. We determined the detection history of individual fish at each receiver (i.e. “re-capture”) line. Fraser River salmon smolts migrated past 2–3 detection lines in the Fraser River (depending on year). There were multiple receiver lines or units in the ocean where they could be detected (with some variation among years), but typical migration routes after ocean entry could not be established because few fish were detected on ocean receivers. As a result, we lumped all ocean receiver detections into the final digit of a fish's detection history sequence, and limited our inferences of survival to the downstream migration phase and not to the early ocean migration (i.e., we disregard any estimates of the confounded parameters φ in the final ocean “segment” and *p* at the final ocean “line”).

Detections of Thompson coho were relatively few on river or ocean lines, when compared to detection data for Thompson spring Chinook salmon (*O. tshawytscha*) and steelhead trout [46], and Cultus Lake sockeye salmon (*O. nerka*) [50]. Therefore, we used information from smolts of other Fraser River populations tagged as part of the POST project to better estimate *p* on river lines. Combining populations and species in the same analysis had the following three advantages: (1) sample sizes of detected coho at receiver stations were often small, so using information from other species can result in more reliable parameter estimates; (2) it allows for a common relationship between model parameters (especially *p*) and environmental covariates like river level or day of year, since this relationship is expected to be consistent across populations; and (3) it allows for a common effect of tag type on *p* across populations. We assume that the same tag type (and therefore acoustic power) passing over a river receiver line around the same time has the same probability of being detected regardless of the species or population from which the tagged smolts originated (apart from run-timing differences between populations; see below). We constructed similar detection histories for steelhead trout and Chinook salmon smolts released in the Thompson watershed, and for sockeye salmon smolts released from Cultus Lake. We used the detection history sequences of individual fish with various mark-recapture models implemented in Program MARK [51] through RMark [52] to estimate φ in-river segments as well as *p* on river lines in each year for each population. For further experimental details about the other Fraser River populations, see Welch et al. [46].

To determine whether survival or detection probabilities were best described as functions of factors such as tag size, river flow, release day, or average travel time, we considered several candidate models. We combined all three years in a detection history dataset and assigned ‘year’, ‘species’ and ‘population’ as group covariates on survival probability estimates. Combining years allowed us to constrain the relative difference in *p* between V7 tags and V9 tags to be consistent (in logit-space) across receiver lines and years (i.e., tag size was an additive covariate). In some models, it also allowed us to maintain a consistent relationship (slope) between either φ or *p* and a model covariate (day of year, river level, or travel time) across lines and years. Although parameter estimates were related through the slopes of such covariates in some models, the intercepts were permitted to differ, thereby maintaining some independence in parameter estimates among lines, years, species, and populations.

We considered six candidate models for *p*, comparing them with information-theoretic criteria [53]. In this comparison we assumed a common sub-model for φ, where parameter estimates for each segment (‘seg’, or ‘time’ in usual nomenclature) and group (year, species, tag type, population) varied freely, i.e., φ(seg×G). These sub-models of *p* were: (i) *p*(line×G); (ii) *p*(line×year); (iii) *p*(line×year+tag type); (iv) *p*(line×year+tag type+day of year); (v) *p*(line×year+tag type+flow_Mission_); and (vi) *p*(line×year+tag type+flow_Port Mann_). Submodel (i) had freely-varying parameters for each detection line (i.e., replacing ‘time’) and group (along with φ, this is the classic CJS model). All other sub-models also maintained independence between different lines and years, i.e., *p*(line×year…). One of these (ii) assumed no difference in detection probability between V7 and V9 tags while the others (iii–vi) assumed an additive difference that was consistent among years, i.e., *p*(…+tag type…); Table 1). Three of these sub-models took into account the mean day of arrival of a population on a receiver line and assumed that *p* at each line was a function of day-of-year or water level at that particular mean arrival day, thereby allowing for variation in *p* among populations through use of these covariates. Water level (which is correlated with river flow) was measured at either the Mission (near the first receiver line in 2004; Fig. 1) or Port Mann (near the second receiver line in 2005) gauge stations [31]. One of these sub-models involved the day-of-year (iv) of arrival at a line as a covariate, another involved the water level at Mission (v), and a third involved the water level at Port Mann at the mean arrival time at a line (vi). Thus, these three sub-models (iv–vi) involved relationships with additive covariates that constrained the effect of the covariate on *p* to be similar among populations and species, but *p* estimates still differed by way of populations and species having their own particular values of the covariate at each receiver station.

**Table 1:** Model selection results for recaptures-only survival (φ) and detection probability (*p*) estimates.^a^

Model	*np*	−2·ln(*L*)	QAIC_c_	ΔQAIC_c_	Akaike weight
Detection probability sub-models^b^					
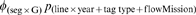 d	94	3 502.7	2 384.8	0.0	0.98
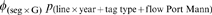 d	94	3 515.4	2 392.8	8.0	0.02
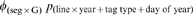 d	94	3 528.5	2 400.9	16.1	0.00
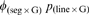	160	3 379.4	2 454.8	70.0	0.00
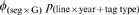	93	3 633.1	2 464.1	79.3	0.00
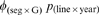	92	3 688.7	2 496.7	111.9	0.00
Survival probability sub-models^c^					
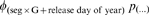	95	3 495.6	2 382.6	0.0	0.65
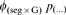	94	3 502.7	2 384.8	2.3	0.21
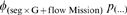 e	95	3 502.7	2 387.0	4.4	0.07
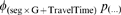	95	3 502.7	2 387.0	4.4	0.07

a Quantities shown are the number of parameters (*np*), log-likelihoods, QAIC_c_ values (adjusted for small sample sizes and extra-binomial variation with 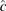 = 1.60), and Akaike weights.

b Sub-models for *p* are compared while the fully time- (“seg”) and group-varying CJS sub-model for φ is held constant, φ_(seg×G)_ . Groups consist of separate combinations of species, population, tag type, and year.

c Sub-models for φ are compared while the sub-model for *p* is held constant at the best model from the above model set, *p*_(line×year+tag type+flow Mission)_.

d Flow covariate terms specify that *p* estimates on receiver lines are dependent upon the river water level measured at either Mission or Port Mann guage stations [31] or upon day of year. See text.

e The flow covariate term specifies that φ estimates in river segments are dependent upon the river water level measured at the Mission guage station at the start of each segment. See text.

After using model selection methods to identify the best sub-model for *p*, we held this sub-model fixed in order to compare four candidate sub-models of φ: (a) φ(seg×G); (b) φ(seg×G+release day of year); (c) φ(seg×G+flow_Mission_); and (d) φ(seg×G+TravelTime). This two-step process of first comparing hypotheses of *p* sub-models before comparing hypotheses of φ sub-models has been used in several other studies (e.g. [54]). To reduce the effect of incorporating other species and populations into the same dataset on φ estimates of Thompson coho, we maintained independence between groups and segments in all four sub-models, φ(seg×G…). One of these sub-models (a) contained no extra covariates so represented complete independence in φ estimates between groups in each segment of the migration. The other three sub-models involved group covariates that were used to explain some of the among-population variation in survival probabilities in terms of variables specific to each population; these could reveal potential correlates of survival that would not be possible by considering Thompson coho alone. One sub-model (b) involved the average release day-of-year as a covariate on all river segments of the migration (all coho populations had only a single release day each year, but some populations of other species were released at two or more periods). Another sub-model (c) involved the average travel time of the population within each segment. The last sub-model (d) involved the water level at the Mission gauge at the start time of each segment of each population (the time of fish release for the first segment and the mean time of arrival at a receiver line for the second or third segments).

We estimated a variance inflation factor (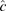) to compensate for extra-binomial variation in estimated probabilities [55]. We estimated 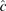 assuming the general CJS model, φ(seg×year), *p*(line×year), using two methods through Program MARK: the deviance ratio bootstrapping method (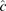 = 1.600) and median-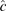 method (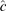 = 1.281±0.037 s.e.) We used the larger value from the bootstrapping routine to be more conservative about the precision of estimated parameters, as these 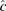 values were used to expand standard errors of real parameter estimates and values in the variance-covariance matrix. Estimated 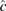 was also used for model comparison, with computed QAIC_c_ values corrected for both extra-binomial variation and small sample sizes.

After the best sub-model for *p* was identified and sub-models for φ were compared, we computed model-averaged parameter estimates for φ in each segment for each population and year. We calculated survivorship estimates from release until the last in-river detection line in each year as the product of segment-specific φ estimates. We used the Delta method to calculate the variance of this product.

## Results

### Model selection

Of the six detection probability sub-models evaluated across all years, the strongest support by far was found in sub-model (v), which involved line- and year-specific estimates with an additive term for tag size and an additive term for river level at the Mission gauge during the mean time of arrival of populations on receiver lines (Table 1). The difference in ΔQAIC_c_ values between this and the next-best sub-model was fairly large (≈8), suggesting little support for this alternative model and essentially no support for any remaining models (ΔQAIC_c_>16) compared with the best sub-model. Detection probability estimates across receiver lines and years (Table 2) therefore varied strongly with both tag size (signal strength) and river level (or flow); lower *p* estimates were associated with the smaller V7 tags and higher water levels (greater flow).

**Table 2:** Detection probability (*p*) estimates (and standard error SE) by tag type and Fraser River array (Line from furthest upstream (1) to furthest downstream (3)).

Year	Tag	Line	*p*	SE
2004	v7	1	1.1%	2.6%
2004	v7	2	7.3%	12.4%
2004	v7	3	100.0%	0.0%
2004	v9	1	72.8%	17.9%
2004	v9	2	95.1%	8.4%
2004	v9	3	100.0%	0.0%
2005	v7	1	39.7%	14.4%
2005	v7	2	43.0%	25.4%
2006	v7	1	48.3%	6.3%
2006	v7	2	49.4%	6.5%
2006	v7	3	55.6%	7.0%

Assuming this best sub-model for *p*, the strongest support among the four survival probability models was found in sub-model (b), which involved a release-day covariate, as measured by ΔQAIC_c_ values (Table 1) and Akaike weights (a proportional measure of support for each model within the model set). The model “beta” coefficient for this day-of-year parameter was significantly less than zero (−0.036; 95% confidence limits: −0.062 to −0.010). Across all river segments and years, populations with later release days therefore tended to have lower downstream survival rates. A moderate level of support (ΔQAIC_c_ values of 2.3–4.4) was still seen in the other three sub-models, however. The classic CJS sub-model without group covariates had 21% support. The sub-model with a covariate of water level at Mission at the mean start time of each segment for each population as well as the sub-model with a mean travel time covariate for each population in each segment each had >7% support within this set of sub-models.

### Detection probability estimates

Survival estimates depend on simultaneously estimated detection probabilities at receiver stations. Estimated detection probabilities of Thompson coho on individual receiver lines in the Fraser River (*p*_i_) ranged widely from 10–85% across receiver lines and years (average of 43%; Table 2). In 2004, V9 tags had higher associated *p*_i_ estimates than V7 tags, permitted by the additive tag size covariate used across populations. Detection probabilities tended to be lower in 2005 and 2006, either due to changed location of receivers or different river conditions at the time of crossing receiver lines. Taking the product of (1-*p*_i_) for all Fraser River lines *i* in each year results in the probability of a smolt crossing all Fraser lines without being detected [56]. This product ranged widely—8%, 67%, and 59% for V7 tags during 2004, 2005 and 2006, respectively, and 2% for V9 tags during 2004. Estimated survival rates account for such imperfect detection probabilities.

### Survival probability estimates

We model-averaged the survival estimates from the four models listed at the bottom of Table 1 to reflect our uncertainty as to which model(s) best fit the observed detection history sequences. As a result of differing Akaike weights, the model-averaged results depend mostly on the model φ(seg×G+day of year), *p*(line×year+tag type+flow_Mission_) (Table 1), but were also influenced by the other three in proportion to these weighting terms. Thompson coho survival rates (and those of other Fraser River populations) are therefore best explained by taking account of the variation among populations and years in the day-of-year of the fish release, and constraining estimated φ parameters to be a function of these days.

**Figure 2:**
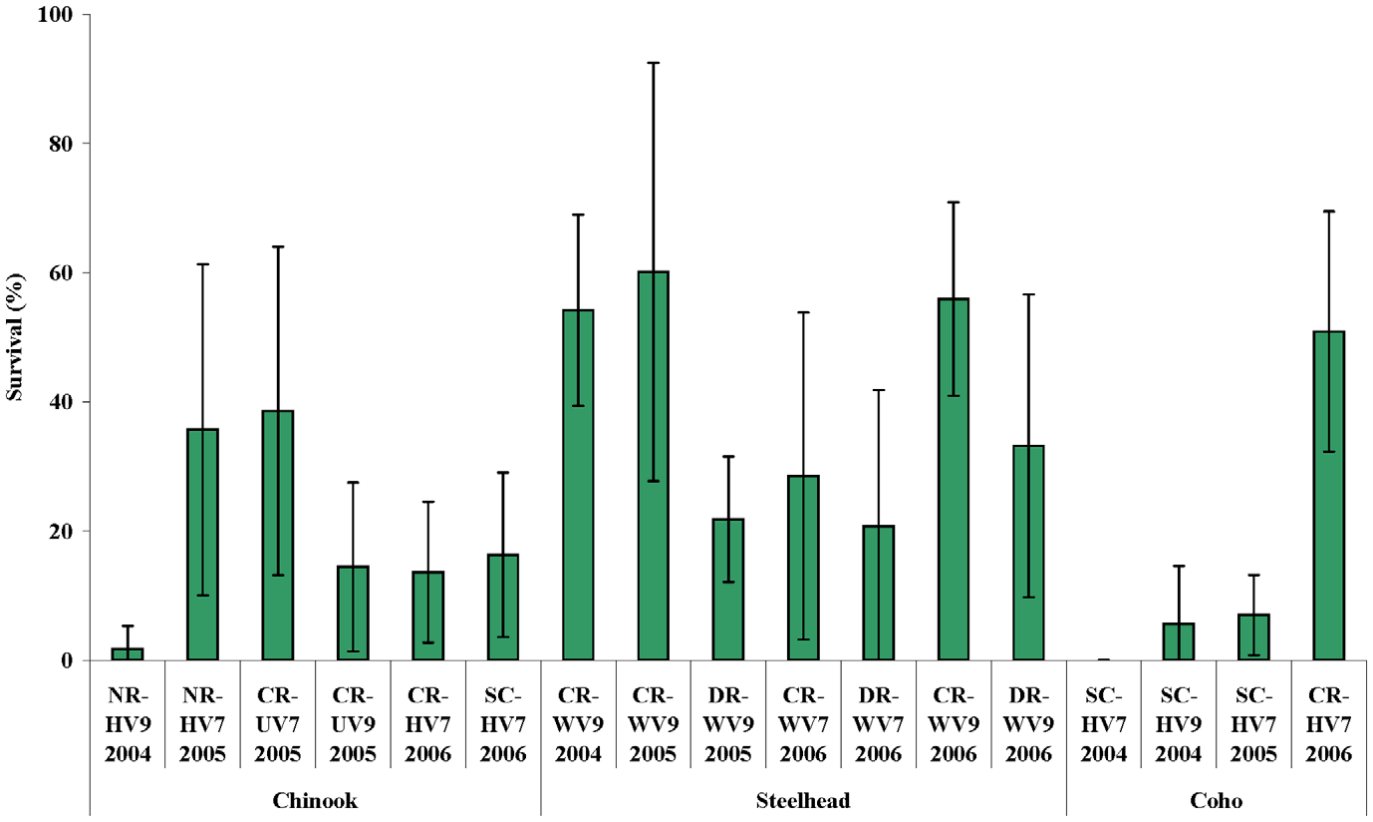
Mark-recapture survival estimates for Thompson River steelhead trout, Chinook and coho salmon smolts. Survival was estimated during the downstream migration from 2004–2006, by tag type. Standard error bars are shown. The smolts were of wild (W), hatchery (H) or unknown (U) origin, from the Coldwater River (CR), Deadman River (DR), Nicola River (NR) and Spius Creek (SC) populations. The same model assumptions were used for Chinook and steelhead as for coho [46].

For two consecutive years, the freshwater survival estimates for Thompson coho smolts reaching the mouth of the Fraser River were low (Fig. 2). Of the 40 fish tagged with V7 (12 individuals) and V9 (28 individuals) tags in 2004, only two tags (one V7 and one V9) were detected in the lower Fraser River. The V7 tag was detected on line 2, 18 days post-release, and the V9 was detected on line 3, 14 days post-release (Tables 2 and 3). None were recorded in the ocean by the POST array. Estimated survival from release to the lowest receiver line in Fraser River was 5.6% (9.0% s.e.) for the V9 group (Fig. 2), but since no fish from the V7 group were detected past the second line, survival to the lowest line was estimated to be 0%. Survival estimates from release to the first and second Fraser River lines were both 14.2% (21.4% s.e.) for the V7 group and both 5.6% (9.0% s.e.) for the V9 group, implying that most mortality was estimated to have occurred before the first detection station (with an additional component of mortality between the second and third stations for the V7 group).

**Table 3:** The number of fish (N) released (rel) and detected (det) at POST listening stations by year and tag type, including the mean fork lengths (FL) and median travel times (T_median_) of the detected fish to the lower Fraser array.

Population	Year	Tag Type	N rel	N det	FL (mm)	T_median_ (Range)
Coldwater	2004	V9-6L	28	1	141	14 (14)
Coldwater	2004	V7-2L	12	1	127	18 (18)
Spius	2005	V7-2L	50	7	127	12 (8–23)
Coldwater	2006	V7-2L	100	24	124	10 (7–19)

During 2005, 50 smolts were tagged with V7 tags, of which four were detected at the Fraser mouth sub-array (all on line 1), 8–23 days post-release (T_median_ = 12 days; Table 3). Two smolts passed through the Fraser array undetected and were subsequently detected at Point Atkinson 16 and 23 days post-release. An additional smolt was recorded in the ocean at the northern Strait of Georgia line one month after release, without having been detected previously in the Fraser (see Table S1 for further details on migratory behaviour). Survival to the mouth of the Fraser River was estimated to be 7.0% (6.2% s.e.; Fig. 2). Survival from release to the first Fraser River line was 45.6% (31.4% s.e.), implying that considerable mortality occurred in both the first and second segments of the migration.

During 2006, 16 of the 100 tagged smolts were detected on line 3 (13 in the north arm and 3 in the south arm) of the lower Fraser 7–19 days post-release (T_median_ = 10 days), and survival was estimated to be 50.9% (18.6% s.e.) from release to each of the three Fraser River lines. In-river mortality, therefore, likely occurred before the first station. Of these 16 fish, three were subsequently recorded in the ocean—one in Burrard Inlet 24 days post-release (in June), and two on the northern Strait of Georgia line 37 and 48 days post-release (in July; Table S1). An additional eight fish were detected in the ocean and not the river. During the summer, one fish was detected in Howe Sound, one in the southern Strait of Georgia, two at Point Atkinson, and four in the northern Strait of Georgia (Table S1).

Due to the low detection rates of Thompson coho on the marine lines and the high delay time between ocean entry and marine detection, no early marine survival estimates could be made. However, coded-wire tagging of smolts released from the Spius Creek Hatchery provided estimated smolt-to-adult survival rates (Doug Turvey, Spius Creek Hatchery, Fisheries and Oceans Canada, Merritt, BC, Canada, *pers. comm.*) During 2004, 84,972 coho smolts were released, of which only 0.43% survived to return. The 2005 releases (n = 41,461) had an even lower overall survival (0.01%). Of the 42,333 smolts released in 2006, 1.52% returned.

## Discussion

The smolt-to-adult survival rates of some salmon populations may depend primarily on the marine phase of their lifecycle (e.g. [2], [14]). However, evidence from this three-year acoustic telemetry study suggests that the low return rates of the endangered Thompson River coho salmon may be strongly affected by mortality during their freshwater out-migration phase. Since the late 1980s return rates of Thompson coho have been consistently lower than predicted, falling below the threshold required for maintaining the demographic and genetic needs of the Management Unit [3], [57]. Although concern was expressed that young out-migrating Thompson coho had poor freshwater survival, no data existed to support this hypothesis [11]. Acoustic monitoring of coho smolts found extremely low freshwater survival rates during 2004 and 2005 (0–7%). Smolt survival was higher during 2006 (51%), which was consistent with the trend observed in hatchery return rates (2004 and 2005: 0.01–0.43%; 2006: 1.52%).

Low freshwater survival rates of Thompson coho smolts during the 2004 and 2005 out-migrations may be the main reason for the extremely low return rates of those year classes. However, the cause of high freshwater mortality in this population remains unknown. The freshwater survival rates of Lower Thompson River steelhead trout and spring Chinook salmon smolts were greater than those of coho during 2004 and 2005 (Fig. 2) [46]. Survival differences between species in the same river system could be the result of many possible factors, including smolt physiology (e.g. overall health, sensitivity to environmental properties) and ecosystem niche (e.g. migration timing, use of habitat, interactions with competitors and predators). If the 2004 and 2005 coho releases had poor health, their predator-avoidance and foraging abilities would have likely been compromised. However, there were no observable signs of ill health in any of the three tagged populations (Doug Turvey, Spius Creek Hatchery, *pers. comm.*) Furthermore, long-term experimental trials to assess survival and tag retention found no evidence of elevated mortality post-surgery [36]. The effects of physical and behavioural differences between species on freshwater survival rates should be investigated further in the Thompson system.

The freshwater out-migration survival of Thompson coho was lower than that of other BC coho populations [45], [58]. River length and habitat quality may play major roles in the survival differences observed between coho populations. Thompson coho smolts must travel over 350 km to reach the ocean, with the first quarter of their journey in the Thompson River, and the remaining part in the Fraser River mainstem. The Fraser River watershed drains approximately one quarter of the land area of BC, with introductions from sewage, agriculture, mines and mills. With over two million people inhabiting the lower Fraser Valley, habitat degradation has also had a detrimental effect on the river and its estuary. Elevated levels of aluminum, iron, zinc, phosphorus, fecal coliform and turbidity observed in the watershed during the spring freshet are of concern, as this is the period when smolts tend to migrate downstream. An assessment of the impact of copper in this system should be carried out immediately, as the minimum detectable limits for monitoring are higher than the upper limits for salmon.

Predation, competition and pollution levels in each river system fluctuate from year to year, and one or more of these factors may have been elevated in the Thompson and Fraser system during 2004 and 2005. Although it seems that the majority of the smolt mortality occurred before the first Fraser receiver line, there was noticeable mortality between the second and third lines during 2004 and 2005. By acoustically monitoring other Fraser coho populations and increasing the receiver-array density in the Fraser River, and in its major tributaries, high-mortality areas can be pinpointed and better understood.

Survival and detection probability estimates of smolt populations were not fully separate in this analysis, primarily because coho detection data alone were too sparse to properly estimate model parameters. Combining detection data for other species in the same dataset improved the estimation of array detection efficiency for the specific types of acoustic tags used in this study, while maintaining partial independence of the coho survival estimates through the use of population and species-specific intercepts. The assumption of a common *p* among populations for the same tag type is reasonable, as detection processes involve tag and receiver characteristics more than species or population characteristics. The assumption of a common effect among species and populations of a covariate (day of release, travel time, river level) on survival probabilities also seems reasonable, although it is possible this effect may be stronger in some species than others. Model-averaging survival probabilities accounted for the uncertainty in which model(s) best fit the detection data, and coho survival estimates were generally robust to the inclusion of those survival covariates that were shared among populations and species. For example, coho survival estimates from release to the river receiver station furthest downstream using the best sub-model (b) with a release day covariate (2004 survival: <0.01 (V7) and 0.07 (V9); 2005: 0.08; 2006: 0.55) were not substantially different from those using the sub-model (a) that involved no covariates on survival (2004 survival: <0.01 (V7) and 0.04 (V9); 2005: 0.06; 2006: 0.43), and did not alter the conclusion that very low downstream survival of coho was observed in both 2004 and 2005.

Freshwater survival estimates reported in this study have several potential sources of bias. The sources can be classified as either positive bias (false survivals e.g. detections from dead fish or tags not actually present), or negative bias (false mortalities e.g. due to tag effects, fish passing by undetected or fish residualising in the upper watershed). Because of the design of the POST architecture, an upper bound on the overall false positive rate for the POST array is estimated to be <0.25% of all detections [59]. While it is possible that dead tagged fish were detected by the river array (e.g. [60]), this seems unlikely given the size of the watershed. Detection probability estimates for each receiver line incorporate negative bias due to missed detections into survival estimate calculations (Table 2, Fig. 2). Because the Fraser array was located near the mouth of the river, the possibility that some coho residualised upstream of the array and remained in freshwater for one or more years post-release before migrating to sea cannot currently be excluded. However, permanent residualisation is rare in coho salmon, and in southern British Columbia the occurrence of coho smolts spending two years in freshwater prior to migrating to sea is generally <1% [33]. While the laboratory tag effects study showed that surgically implanted Thompson River coho smolts had 100% survival to the end of the detection period observed in the field [36], there may be unforeseen tag effects that have not yet been identified in the wild [59], [61]. Differences between hatchery and wild smolts should also be considered when examining the results of this study. Hatchery-reared smolts may be less adapted to surviving in the wild environment [58], [62], [63]. Thus, survival estimates of hatchery smolts may be lower than actual survival rates for wild Thompson coho. Alternatively, the larger hatchery smolts may have been stronger migrants with higher survival than the wild smolts.

While ecosystem dynamics in the Thompson and Fraser Rivers may cause high coho mortality some years (e.g. in 2004 and 2005), during other years coho smolts may reach the Strait of Georgia in higher numbers (e.g. in 2006). Thus, factors influencing the marine survival of the Thompson coho, as well as other southern coho populations, remain a concern [16]. Whereas Fraser River steelhead trout and sockeye salmon migrate out of the Strait of Georgia quickly [46], Fraser coho historically spent their entire marine phase within the strait [27]. However, recent changes to the Strait of Georgia ecosystem may be affecting juvenile coho migratory behaviour and mortality rates [64]. For the conservation of the Thompson River coho salmon and other populations of concern, further investigations into population health, hatchery-rearing effects, habitat quality, and other ecosystem dynamics are needed in both the freshwater and marine environments.

## Supporting Information

Table S1 Post-release detection locations of acoustically tagged Thompson River coho salmon smolts (FL is fork length, NSOG is the northern Strait of Georgia, SSOG is the southern Strait of Georgia).
(0.05 MB DOC)
