# Correction: An Investigation into the Poor Survival of an Endangered Coho Salmon Population

**DOI:** 10.1371/annotation/bb9a1641-47fb-4aff-ba84-f44d8fbe62ab

**Published:** 2010-06-15

**Authors:** Cedar M. Chittenden, Michael C. Melnychuk, David W. Welch, R. Scott McKinley

There was an error in the Competing Interests section. The correct competing interest information is as follows: Dr. David Welch is the president of Kintama Research. Kintama Research is an environmental consultancy that designed and deployed large parts of the current array. Their operating costs partly come from the Gordon and Betty Moore Foundation. The Gordon and Betty Moore Foundation was not directly involved in the study design, analysis, or writing of the paper, nor the decision to submit the manuscript for publication. This does not alter the authors' adherence to all the *PLoS ONE* policies on sharing data and materials, as detailed online in the *PLoS ONE* Author Guidelines. 

