# Correction: An Investigation into the Poor Survival of an Endangered Coho Salmon Population

**DOI:** 10.1371/annotation/fe4cfa6b-d2d3-4c3c-bca0-492effe02787

**Published:** 2010-06-15

**Authors:** Cedar M. Chittenden, Michael C. Melnychuk, David W. Welch, R. Scott McKinley

There was an error in the Funding section. The correct funding information is as follows: Funding for this work was provided by the Census of Marine Life, the Gordon and Betty Moore Foundation, and the Pacific Salmon Foundation. The Pacific Ocean Shelf Tracking project (POST) acoustic receiver arrays were designed and deployed by Kintama Research Corp. None of the first three organizations were directly involved in the study design, analysis, or writing of the paper, nor the decision to submit the manuscript for publication. This work is a contribution to the Census of Marine Life.

