# Supplementary material for: An Investigation into the Poor Survival of an Endangered Coho Salmon Population
Source: PLoS One. 2010 May 28;5(5):e10869. doi: 10.1371/journal.pone.0010869 (PMC2878331; doi:10.1371/journal.pone.0010869)
Supplement: Table S1 — Post-release detection locations of acoustically tagged Thompson River coho salmon smolts (FL is fork length, NSOG is the northern Strait of Georgia, SSOG is the southern Strait of Georgia). (0.05 MB DOC) [file pone.0010869.s001.doc]

Table S1. Post-release detection locations of acoustically tagged Thompson River coho salmon smolts (FL is fork length, NSOG is the northern Strait of Georgia, SSOG is the southern Strait of Georgia).

| Year | Tag Type | FL (mm) | Location (Days Post-Release) |
| --- | --- | --- | --- |
| 2004 | V9-6L | 141 | Fraser R. (14) |
| 2004 | V7-2L | 127 | Fraser R. (18) |
| 2005 | V7-2L | 125 | Fraser R. (8) |
| 2005 | V7-2L | 123 | Fraser R. (12) |
| 2005 | V7-2L | 128 | Fraser R. (12) |
| 2005 | V7-2L | 120 | Pt. Atkinson (16) |
| 2005 | V7-2L | 139 | Fraser R. (23) |
| 2005 | V7-2L | 125 | Pt. Atkinson (23) |
| 2005 | V7-2L | 130 | NSOG (34-41) |
| 2006 | V7-2L | 129 | Fraser R. (7) |
| 2006 | V7-2L | 131 | Fraser R. (8) |
| 2006 | V7-2L | 132 | Fraser R. (9) |
| 2006 | V7-2L | 132 | Fraser R. (9), NSOG (47) |
| 2006 | V7-2L | 130 | Fraser R. (9) |
| 2006 | V7-2L | 134 | Fraser R. (9) |
| 2006 | V7-2L | 127 | Fraser R. (9) |
| 2006 | V7-2L | 128 | Fraser R. (9) |
| 2006 | V7-2L | 129 | Fraser R. (10) |
| 2006 | V7-2L | 132 | Fraser R. (10), Pt. Atkinson (13), NSOG (38) |
| 2006 | V7-2L | 133 | Fraser R. (11) |
| 2006 | V7-2L | 132 | Fraser R. (11), Burrard Inlet (24) |
| 2006 | V7-2L | 126 | Fraser R. (15) |
| 2006 | V7-2L | 125 | Fraser R. (16) |
| 2006 | V7-2L | 130 | Fraser R. (16) |
| 2006 | V7-2L | 126 | Pt. Atkinson (17-21) |
| 2006 | V7-2L | 130 | Fraser R. (19) |
| 2006 | V7-2L | 127 | Pt. Atkinson (19) |
| 2006 | V7-2L | 129 | Howe Sound (25) |
| 2006 | V7-2L | 132 | NSOG (36) |
| 2006 | V7-2L | 131 | NSOG (37) |
| 2006 | V7-2L | 125 | NSOG (58) |
| 2006 | V7-2L | 129 | SSOG (67-121) |
| 2006 | V7-2L | 130 | NSOG (102) |
